# A Novel Impedance Micro-Sensor for Metal Debris Monitoring of Hydraulic Oil

**DOI:** 10.3390/mi12020150

**Published:** 2021-02-03

**Authors:** Hongpeng Zhang, Haotian Shi, Wei Li, Laihao Ma, Xupeng Zhao, Zhiwei Xu, Chenyong Wang, Yucai Xie, Yuwei Zhang

**Affiliations:** Marine Engineering College, Dalian Maritime University, Dalian 116026, China; zhppeter@dlmu.edu.cn (H.Z.); dmuliwei@dlmu.edu.cn (W.L.); 15641167110@163.com (L.M.); zhaoxp789@163.com (X.Z.); xuzhiwei201809@163.com (Z.X.); wangcy_dlmu@163.com (C.W.); Xyc86418332@163.com (Y.X.); vivi6346@sina.com (Y.Z.)

**Keywords:** condition monitoring, metal debris, impedance micro-sensor, hydraulic oil

## Abstract

Hydraulic oil is the key medium for the normal operation of hydraulic machinery, which carries various wear debris. The information reflected by the wear debris can be used to predict the early failure of equipment and achieve predictive maintenance. In order to realize the real-time condition monitoring of hydraulic oil, an impedance debris sensor that can detect inductance and resistance parameters is designed and studied in this paper. The material and size of wear debris can be discriminated based on inductance-resistance detection method. Silicon steel strips and two rectangular channels are designed in the sensor. The silicon steel strips are used to enhance the magnetic field strength, and the double rectangular detection channels can make full use of the magnetic field distribution region, thereby improving the detection sensitivity and throughput of the sensor. The comparison experiment shows that the coils in series are more suitable for the monitoring of wear debris. By comparing and analyzing the direction and the presence or absence of the signal pulses, the debris sensor can detect and distinguish 46 µm iron particles and 110 µm copper particles. This impedance detection method provides a new technical support for the high-precision distinguishing measurement of metal debris. The sensor can not only be used for oil detection in the laboratory, but also can be made into portable oil detection device for machinery health monitoring.

## 1. Introduction

As the key working medium of the mechanical system, oil can not only be used to transfer energy, but also play the role of lubrication, cooling, oxidation inhibition, vibration reduction and equipment life extension. The cleanliness of the oil has an important impact on the reliability, sensitivity, stability, working efficiency and service life of the mechanical equipment. According to statistics, more than 75% mechanical system failures are caused by oil pollution [1]. As one of the main oil contaminants, the size and shape of metal debris can reflect the wear characteristics of components [2]. The oil debris monitoring can ensure that the oil pollution degree is within the acceptable health range of the system, which can effectively prevent equipment failure and machine damage accidents [3,4]. Currently, the detection of hydraulic oil contaminants is mainly divided into offline detection and online monitoring. Offline detection is mainly based on ferrography and spectroscopy analysis in the laboratory [5,6], which can obtain detailed information about the material, size, shape and concentration of contaminants. However, because the offline detection process involves sampling, inspection, laboratory detecting, data analysis, etc., there are shortcomings such as high cost, long cycle, and the inability to reflect the real-time status of hydraulic oil. Online monitoring is a real-time detection method based on different principles of sensor, which mainly includes optical method [7,8], ultrasonic method [9,10], inductance method [11,12] and capacitance method [13,14]. Among them, the inductance method can distinguish between ferromagnetic and non-ferromagnetic metal debris and has the advantages of simple structure and reliable performance.

Recently, inductive debris sensors with various structures are proposed. The MetalSCAN developed by GasTOPS is applied on condition assessment on the rolling element bearings in aircraft [15], which can detect 100 µm ferromagnetic and 405 µm non-ferromagnetic spherical particles by the debris sensor with triple-coil structure. By the filtering technique [16,17], the unbalance compensation circuit [18], the series-parallel resonant circuit [19] and other methods, the detection capacity of the triple-coil debris sensor is improved. Ding et al. [20] designed a new structure of online debris sensor to reduce the interferences of the noise and the vibration, where the inductive coil is located in a radial magnetic field generated by the upper and lower planar coils. Ren et al. [21] introduced an inductive sensor with multiple sensing coils based on one energizing coil to improve the detection sensitivity. Xiao et al. [22] presented an inductive debris sensor based on a high-gradient magnetic field, the magnetic field produced by the excitation coil driven by a constant current, and the induction coil on the flow path is used for monitoring. Wang et al. [23] proposed a debris sensor with dual excitation sources structure to improve magnetic field uniformity for higher accuracy detection result. After adding the shielded metal shell, the above-mentioned debris sensor is installed on the oil circuit or oil circuit bypass of the mechanical equipment. However, affected by the working environment of the mechanical system, the detection sensitivity of the monitoring device installed in the oil circuit is limited. Generally, it can only detect ferromagnetic debris with the size greater than 100μm. Since the large diameter of the sensor detection channel, the signal caused by two debris particles cannot be separated when their distance is less than a certain range.

With the development of micro-machining technology, inductive sensors with small size detection channels have been developed to accurately measure debris and achieve particle counting. Du et al. [24] demonstrated an inductive device based on the Coulter counting principle, which can detect iron and copper particles ranging in size from 50 to 125 μm. Ma et al. [25] investigated double-wire solenoid coil sensor, which uses mutual inductance to detect smaller size metal debris. Liu et al. [26] proposed a micro inductive sensor with magnetic powder surrounded, which can achieve better detection effect by increasing concentration of magnetic powder. Zeng et al. [27] presented a micro impedance sensor with two coils, and the detection sensitivity was improved by focusing the magnetic field [28]. These micro-sensors can be used as the core component of portable oil detection devices for real-time sampling monitoring on site.

This paper reports a method of oil debris monitoring using an impedance debris sensor. The sensitivity of sensor can be enhanced by adding silicon steel strips and connecting coils in series, and the throughput can be improved by the design of double rectangular channels. By detecting and analyzing the inductance and resistance data, the sensor can obtain the debris information about material, size and number of wear debris. The sensor can not only be used in the laboratory, but also can be made into portable oil detection device for machinery health monitoring.

## 2. Sensor Design

**Figure 1:**
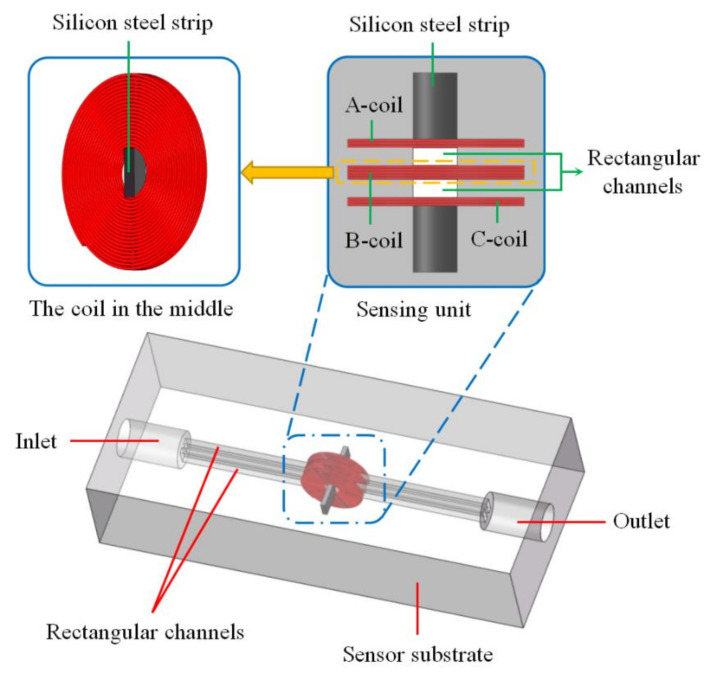
The structure of impedance debris sensor.

As shown in Figure 1, the debris sensor mainly includes a channel inlet, double rectangular channels, a sensing unit, a channel outlet and a sensor substrate. The sensing unit is mainly composed of three planar coils placed in parallel, the distance between the coils is 500 µm, and the central hole of each coil is added with silicon steel strip. The coil is made of 70 µm enameled wire, each layer is 20 turns, the coil inner diameter is 900 µm, and the coil outer diameter is about 3.8 mm. The coil in the middle is 5 layers, and the coils on both sides are 3 layers. The thickness of the silicon steel strip is 300 µm and the width is 800 µm. The length of the silicon steel strip in the middle is 320 µm, and the length of the silicon steel strips on both sides is 1.5 mm. The rectangular channel passes between the two coils and is close to the coils. The diameter of the channel inlet and outlet is 5 mm. In order to observe the particles in the channels, the sensor substrate is cast with transparent PDMS. The length, width and height of the substrate are 8 cm, 2.5 cm and 1.5 cm, respectively.

## 3. Detection Principle

The coil is excited by alternating current, and an alternating magnetic field is induced around the coil. When the metal wear debris passes through the sensing unit, the coil impedance will change due to electromagnetic induction. Based on previous studies [29], the coil impedance *Z* can be calculated by

(1) $$Z=\frac{j\omega n_{c}}{I}\int_{\tau }\left(A+\Delta A\right)\;d\tau$$

Here, I is the current in the coil, ω is the angular frequency of excitation alternating current, $n_{c}$ is the current density, A is the magnetic vector potential, τ is the coil volume, and $j^{2}=-1$.

ΔA is the change of magnetic vector potential caused by a particle.

(2) $$\begin{array}{l} \Delta A=\frac{\left(-ja^{2}\omega \mu_{r}\mu_{o}\sigma -2\mu_{r}-1\right)\sin\left(a\sqrt{-j\omega \mu_{r}\mu_{o}\sigma }\right)+a\sqrt{-j\omega \mu_{r}\mu_{o}\sigma }\left(2\mu_{r}+1\right)\cos\left(a\sqrt{-j\omega \mu_{r}\mu_{o}\sigma }\right)}{\left(-ja^{2}\omega \mu_{r}\mu_{o}\sigma +\mu_{r}-1\right)\sin\left(a\sqrt{-j\omega \mu_{r}\mu_{o}\sigma }\right)-a\sqrt{-j\omega \mu_{r}\mu_{o}\sigma }\left(\mu_{r}-1\right)\cos\left(a\sqrt{-j\omega \mu_{r}\mu_{o}\sigma }\right)}\\ \;\;\;\;\;\;\;\;\frac{a^{3}}{2}B_{p}\times \frac{r-r_{p}}{{\left|r-r_{p}\right|}^{3}} \end{array}$$

Here, a is the radius of particle, $\mu_{r}$ is the relative permeability of particle, $\mu_{o}$ is the permeability of vacuum, σ is the conductivity of particle, $B_{p}$ is the magnetic flux density of the excitation magnetic field, r is the arbitrary position vector of coil, and $r_{p}$ is the position vector of the particle center.

The coil impedance is divided into the self-inductance and AC resistance of the coil. The AC resistance of coil [30] is composed of the DC resistance $R_{dc}$, skin-effect resistance $R_{s}$, and proximity-effect resistance $R_{p}$.

(3) R=Rdc+Rs+Rp=ReZ

After adding silicon steel strip, the self-inductance of the coil *L* is

(4) L=ImZω

Considering multiple coils, the equivalent impedance of the sensor is

(5) $$Z_{eq}=R_{eq}+j\omega L_{eq}$$

$R_{eq}$ is the equivalent AC resistance, $L_{eq}$ is the equivalent inductance.

**Figure 2:**
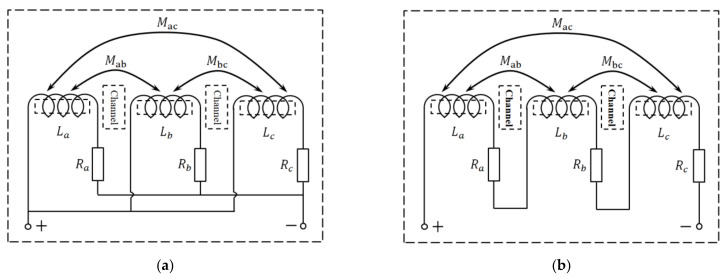
Coil connection in the same direction: (**a**) parallel connection; (**b**) series connection.

In order to ensure that the magnetic fields generated by the coils do not cancel each other, the three coils can be connected in series in the same direction and in parallel in the same direction, as shown in Figure 2. Therefore, the self-inductance of the A-coil, B-coil and C-coil is $L_{a}$, $L_{b}$ and $L_{c}$, respectively. The mutual inductance between A-coil and B-coil is $M_{ab}$, the mutual inductance between A-coil and C-coil is $M_{ac}$, and the mutual inductance between B-coil and C-coil is $M_{bc}$. The AC resistance of the A-coil, B-coil and C-coil is $R_{a}$, $R_{b}$ and $R_{c}$, respectively.

As shown in Figure 2a, the parallel equivalent inductance of the coils is

(6) $$L_{Peq}=\frac{\left|\begin{array}{ccc} L_{a} & M_{ab} & M_{ac}\\ M_{ab} & L_{b} & M_{bc}\\ M_{ac} & M_{bc} & L_{c} \end{array}\right|}{\left|\begin{array}{ccc} 1 & M_{ab} & M_{ac}\\ 1 & L_{b} & M_{bc}\\ 1 & M_{bc} & L_{c} \end{array}\right|+\left|\begin{array}{ccc} L_{a} & 1 & M_{ac}\\ M_{ab} & 1 & M_{bc}\\ M_{ac} & 1 & L_{c} \end{array}\right|+\left|\begin{array}{ccc} L_{a} & M_{ab} & 1\\ M_{ab} & L_{b} & 1\\ M_{ac} & M_{bc} & 1 \end{array}\right|}\;$$

The parallel equivalent AC resistance of the coils is

(7) $$R_{Peq}=\frac{R_{a}R_{b}R_{c}}{R_{a}R_{b}+R_{a}R_{c}+R_{b}R_{c}}\;$$

As shown in Figure 2b, the series equivalent inductance of the coil is

(8) $$L_{Seq}=L_{a}+L_{b}+L_{c}+2M_{ab}+2M_{ac}+2M_{bc}\;$$

The series equivalent AC resistance of the coils is

(9) $$R_{Seq}=R_{a}+R_{b}+R_{c}\;$$

**Figure 3:**
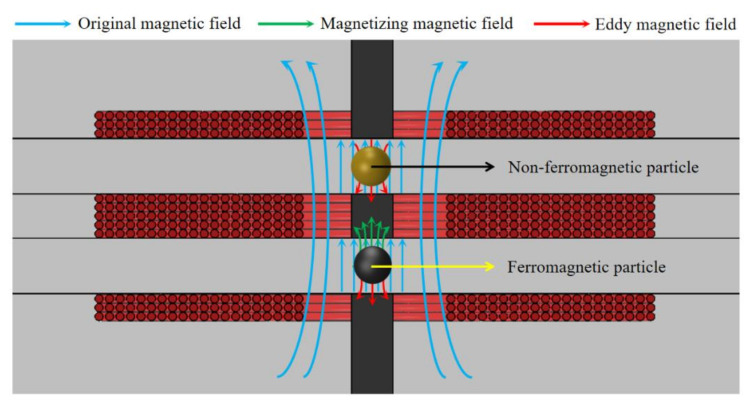
Schematic diagram of sensor detection principle.

As shown in Figure 3, the metal particles in alternating magnetic field will produce a magnetization effect and an eddy current effect. As paramagnetic material, the ferromagnetic wear debris has stronger magnetization effect, generating a co-directional magnetic field (part of the magnetizing magnetic field is canceled by the eddy magnetic field) as a whole to enhance the original magnetic field, thereby the inductance of the coils is enhanced. As diamagnetic material, non-ferromagnetic wear debris has strong eddy current effect, which can generate a reverse magnetic field to weaken the original magnetic field, thereby the inductance of the coils is decreased. When the metal wear debris passes through the coils, it will affect the original skin effect and proximity effect, thereby increasing the AC resistance of the coils and generating resistance pulse.

Equations (1) and (2) show that the excitation frequency also has a greater impact on the detection result of the sensor. The eddy current inside the metal particle will increase with the excitation frequency [31,32]. Moreover, the eddy current changes the original skin effect and proximity effect greatly. In ferromagnetic particles, the direction of magnetizing field is opposite to that of eddy magnetic field. So, for ferromagnetic particle, the inductance change decreases and the resistance change increases with frequency. For non-ferromagnetic particle, the changes in inductance and resistance increase significantly with the frequency. Considering that the inductive sensor has a stronger detection capability for ferromagnetic particles [33], high-frequency excitation should be selected to detect non-ferromagnetic debris with smaller sizes for the comprehensive detection effect of the sensor.

## 4. Impedance Detection System

**Figure 4:**
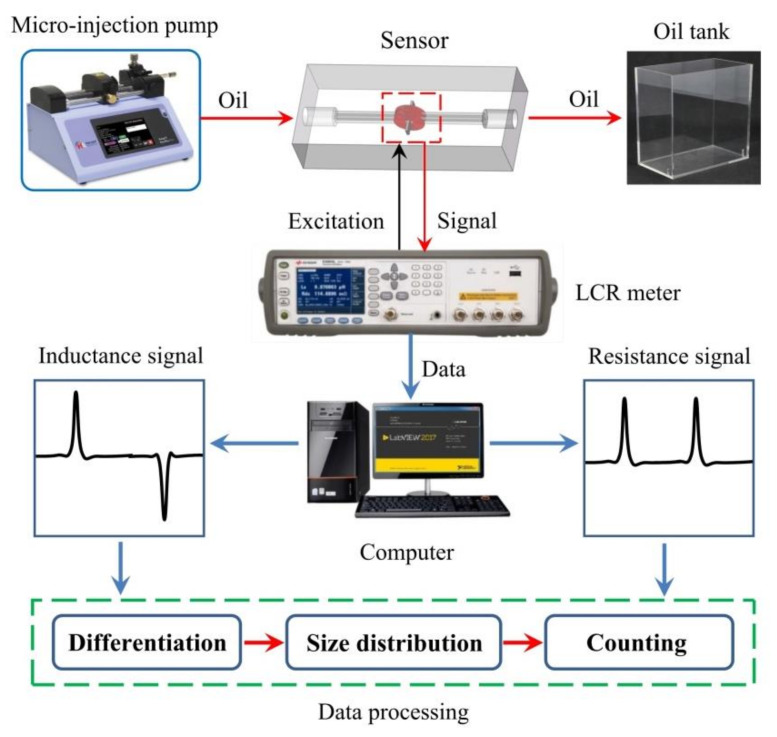
Impedance detection system.

In order to test the performance of the impedance debris sensor, an impedance detection system was established in the laboratory, as shown in Figure 4. The detection system is mainly composed of a micro-injection pump (Harvard pump 11 plus), impedance debris sensors, a microscope (Nikon AZ100), an impedance analyzer (Agilent E4980A) and a computer installed with LabVIEW software and a data analysis counting program.

The microscope is used to measure the size of particles and observe the material of particles in the channel. The micro-injection pump can drive the oil sample at a constant speed, the selected particles can reciprocate through the detection region by adjusting the injection direction. The impedance analyzer can apply alternating current excitation to the coils and obtain the coils’ impedance value in real time. LabVIEW program can transfer data to the computer for storage and display. The data analysis and counting program can distinguish the particle materials, measure the size, and achieve counting by calculating the signal amplitude, number and direction.

## 5. Experiments

**Figure 5:**
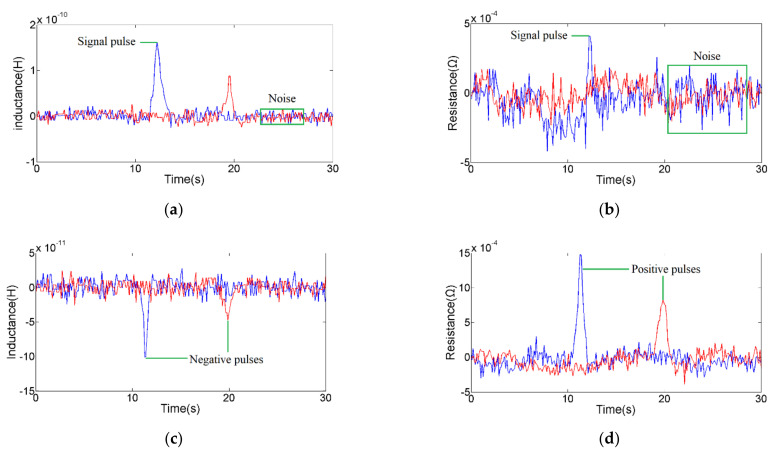
Detection results of coils in parallel; red signal is the result of the sensing unit without silicon steel strips, blue signal is the result of the sensing unit with silicon steel strips: (**a**) inductance pulse of 93 μm iron particle; (**b**) resistance pulse of 93 μm iron particle; (**c**) inductance pulse of 186 μm copper particle; (**d**) resistance pulse of 186 μm copper particle.

**Figure 6:**
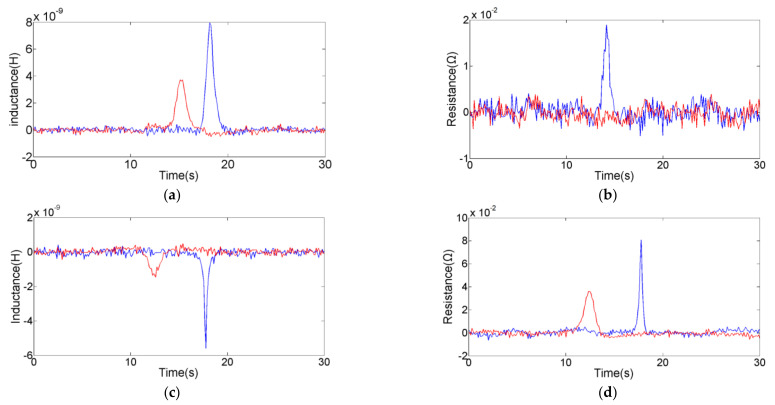
Detection results of coils in series; red signal is the result of the sensing unit without silicon steel strips, blue signal is the result of the sensing unit with silicon steel strips: (**a**) inductance pulse of 93 μm iron particle; (**b**) resistance pulse of 93 μm iron particle; (**c**) inductance pulse of 186 μm copper particle; (**d**) resistance pulse of 186 μm copper particle.

An impedance debris sensor with two sensing units (one has silicon steel strips, another has none) was used in the experiment. In order to prepare the oil sample for the experiment, the 1 mg iron particles and 1 mg copper particles were mixed with 100 mL of hydraulic oil (The Great Wall L-HM 46, Sinopec Lubricant Co., Ltd., Beijing, China). The range of excitation parameters provided by the impedance analyzer is 0–2 V, 0–2 MHz. According to the analysis results of the theoretical part, the excitation parameters were set to 2.0 V, 2.0 MHz for the exploration of the sensor performance. Previous studies [34] have shown that the particles pass slowly through the magnetic field, and the eddy current and magnetization are sufficient. However, the flow should not be set too low for the detection efficiency. The flow rate should be set at 200–500 μL/min in this sensor. Therefore, the flow rate of the micro-injection pump was set to 400 μL/min. The oil sample containing metal debris was injected into the channel by micro-injection pump. Under the microscope, 93 μm iron spherical debris and 186 μm copper spherical debris were selected and were reciprocating through the sensing units for comparative experiments. Then, the selected particles were detected in parallel and series to explore the influence of the coil connection method on the detection results. Shown in Figure 5 and Figure 6 are the inductance and resistance signal diagrams. The red signal is the detection result of the sensor without silicon steel strips, and the blue signal is the detection result of the sensor with silicon steel strips.

In Figure 5 and Table 1 and Table 2, the signal-to-noise ratio (SNR) is usually calculated as the ratio of the pulse value to the standard deviation of the noise. The pulse amplitude is in the form of absolute value to characterize the signal strength. The direction of pulse is introduced to judge the debris material. As shown in Figure 5, the pulse directions from different material particles are different. It is easy to find that the addition of silicon steel strips significantly enhances the detection accuracy of the sensor, whether in series or parallel. For 93 μm iron particle, the amplitude of series inductance pulse increased from 4.28 × 10^−9^ H to 7.64 × 10^−9^ H; the amplitude of parallel inductance pulse increased from 9.40 × 10^−11^ H to 1.72 × 10^−10^ H; there was no resistance pulse without silicon steel strips, but the series resistance pulse (the amplitude is 1.65 × 10^−2^ Ω) and the parallel resistance pulse (the amplitude is 4.70 × 10^−4^ Ω) were generated after adding silicon steel strips. For 186 μm copper particle, the amplitude of series inductance increased from 1.88 × 10^−9^ H to 5.63 × 10^−9^ H; the amplitude of parallel inductance increased from 5.0 × 10^−11^ H to 1.05 × 10^−10^ H; the amplitude of series resistance increased from 3.73 × 10^−2^ Ω to 8.03 × 10^−2^ Ω; and the amplitude of parallel resistance increased from 7.4 × 10^−4^ Ω to 1.69 × 10^−3^ Ω. Under the action of silicon steel strips, the signal noise remains unchanged, while the inductance and resistance pulse amplitudes of metal debris increase significantly. The main reason is that the magnetic strength of the detection region has been enhanced by the magnetization of the silicon steel strips, so that the impedance change caused by metal debris is more obvious.

In comparison experiments of coils in series and parallel, the amplitude of the inductance and resistance pulses obtained in series is much larger than that obtained in parallel. When the coils are connected in series, the basic inductance of the sensor with silicon steel strips is 4.70 × 10^−5^ H, the inductance signal noise is 5.0 × 10^−10^ H; the basic resistance is 59.39 Ω, and the resistance signal noise is 5.0 × 10^−3^ Ω; the SNR of the inductance pulse obtained by 93 μm iron particle is 15.28; the SNR of the inductance pulse obtained by 186 μm copper particle is 11.26; the SNR of the resistance pulse obtained by 93 μm iron particle is 3.30; the SNR of the resistance pulse obtained by 186 μm copper particle is 16.06. When the coils are connected in parallel, the basic inductance of the sensor with silicon steel strip is 2.60 × 10^−6^ H, the inductance signal noise is 3.0 × 10^−11^ H; the basic resistance is 3.80 Ω, and the resistance signal noise is 3.0 × 10^−4^ Ω; the SNR of the inductance pulse obtained by 93 μm iron particle is 5.73; the SNR of the inductance pulse obtained by 186 μm copper particle is 3.50; the SNR of the resistance pulse obtained by 93 μm iron particle is 1.57; the SNR of the resistance pulse obtained by 186 μm copper particle is 5.63. For the same particle, the SNR obtained by the coils in series is larger than that coils in parallel. High SNR represents high detection sensitivity, so the coils in series are more suitable for the monitoring of wear debris.

**Figure 7:**
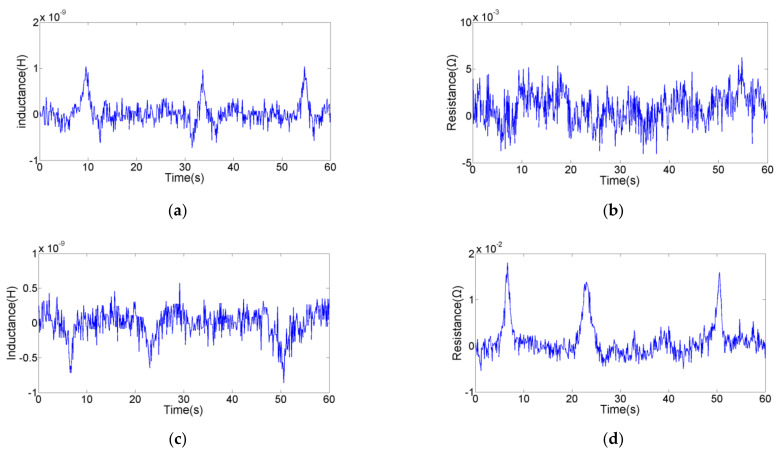
The floor level of inductance detection (with silicon steel strips): (**a**) inductance signal of 46 µm iron particle; (**b**) resistance signal of 46 µm iron particle; (**c**) inductance signal of 125 µm copper particle; (**d**) resistance signal of 125 µm copper particles.

**Figure 8:**
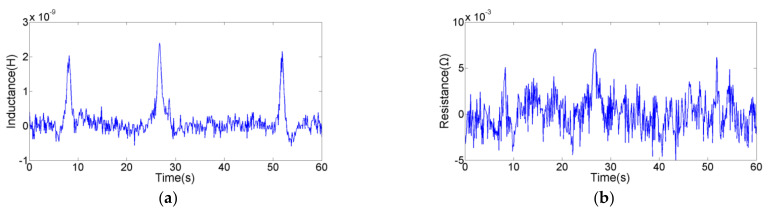

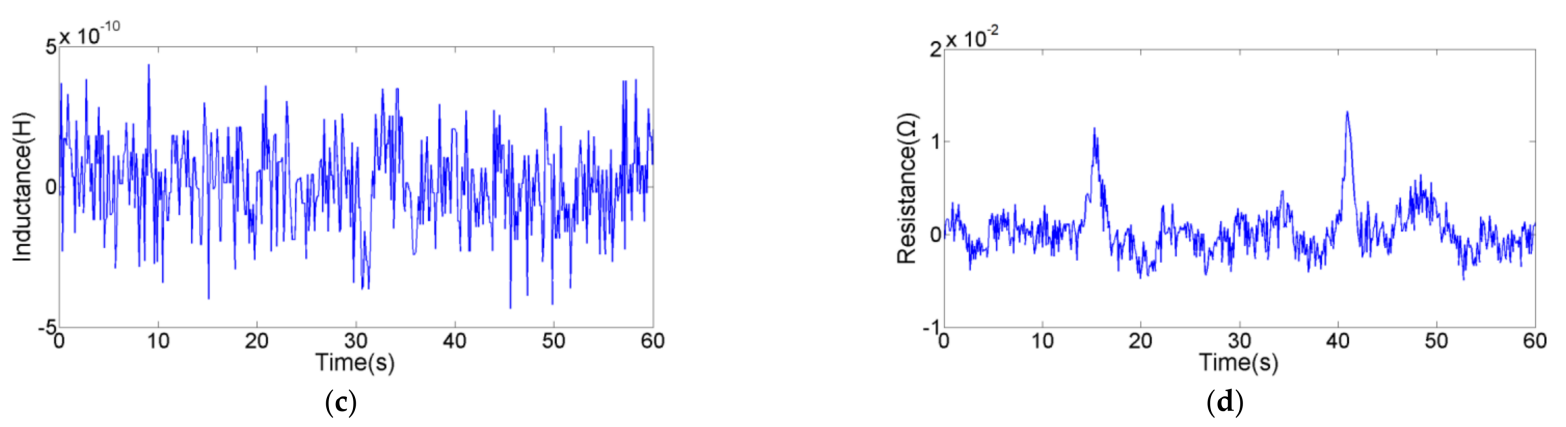
The floor level of resistance detection (with silicon steel strips): (**a**) inductance signal of 59 µm iron particle; (**b**) resistance signal of 59 µm iron particle; (**c**) inductance signal of 110 µm copper particle; (**d**) resistance signal of 110 µm copper particles.

The detection sensitivity in series is higher, and the impedance debris sensor can collect the inductance and resistance signals. In order to test the comprehensive detection ability, we explored the floor level of different parameters for different metal particles used the sensor with silicon steel strips in series. As shown in Figure 7 and Figure 8, the inductance parameter can detect 46 μm iron particle and 125 μm copper particle; the resistance parameter can detect 59 μm iron particle and 110 μm copper particle. This indicates that the inductance parameter can detect smaller ferromagnetic particles, and the resistance parameter can detect smaller non-ferromagnetic particles. Previous studies [35] have shown that the material of wear debris can be judged according to the direction and the presence or absence of signal pulses. In addition, the detection results based on the two parameters are compared to obtain accurate size information. Therefore, the impedance sensor designed in this paper can effectively detect and distinguish 46 µm iron particle and 110 µm copper particle.

**Figure 9:**
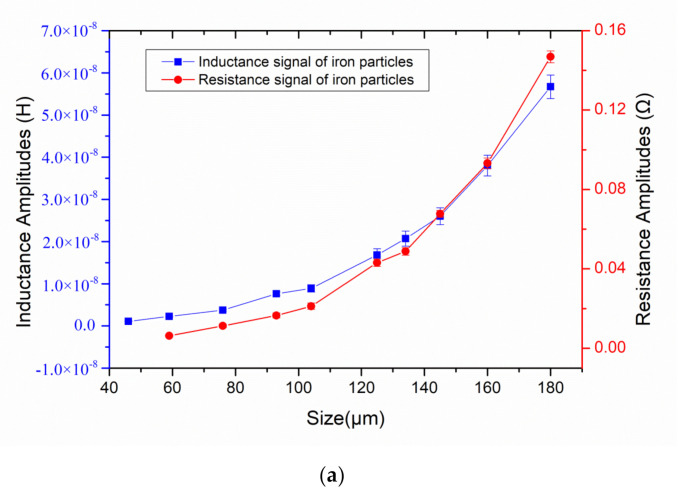

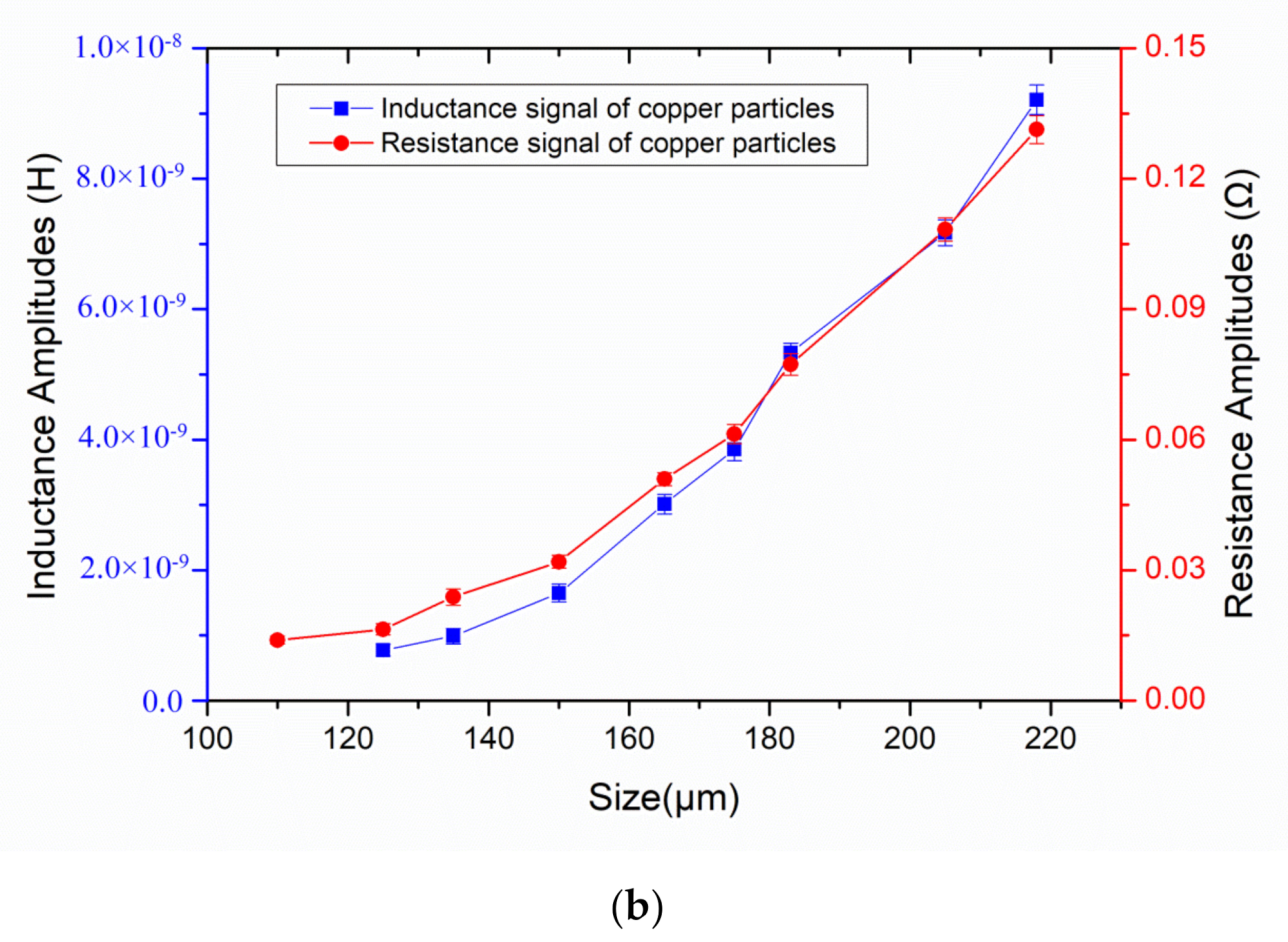
The inductance pulse amplitudes and the resistance pulse amplitudes of metal particles with different diameters. (**a**) detection results of iron particles; (**b**) detection results of copper particles.

In order to obtain the relationship between the particle size and the pulse amplitude, the iron particles and copper particles with different sizes were detected in laboratory. As shown in Figure 9a, the resistance and inductance pulse amplitudes of iron particles increase nonlinearly with the size, which is because the magnetization effect of iron particles enhances nonlinearly. As shown in Figure 9b, the resistance and inductance pulse amplitudes of copper particles increase nonlinearly with the size, which is because the eddy current of copper particles enhances nonlinearly. In the same size metal debris, the inductance amplitude of iron particles is much stronger than that of copper particles, and the resistance amplitude of copper particles is much stronger than that of iron particles. This is because the magnetization effect of iron particles is much stronger than that of copper particles, and the eddy current effect of copper particles is much stronger than that of iron particles. The result of inductance detection mainly depends on the magnetization effect, while the result of resistance detection mainly depends on the eddy current effect. A database on the corresponding characteristics of wear debris and impedance pulse can be established for mechanical equipment health monitoring.

## 6. Conclusions

An impedance debris sensor capable of detecting inductance and resistance parameters is demonstrated. The sensor is designed with three planar coils, silicon steel strips inserted into the coil inner holes and double rectangular channels. Silicon steel strips are used to enhance the magnetic field strength in the detection region, thereby improving the detection sensitivity. Double rectangular detection channels can make full use of the magnetic field distribution region, thereby improving the detection throughput. In the comparison experiments of coils in series and parallel, the amplitude of the inductance and resistance pulses obtained in series is much larger than that obtained in parallel, and the SNR of the series pulses is greater than that of the parallel pulses. When the coils are connected in series, the sensor has higher detection sensitivity and is more suitable for the monitoring of wear debris. The inductance parameter can detect 46 µm iron particles and 125 µm copper particles, and the resistance parameter can detect 59 µm iron particles and 110 µm copper particles. The sensor can discriminate the material and size of wear debris based on inductance and resistance detection results. By analyzing the direction and the presence or absence of signal pulses, the impedance debris sensor can detect and distinguish 46 µm iron particles and 110 µm copper particles. Impedance detection method will provide technical support for high-precision identification and measurement of metal debris. However, the detection sensitivity of the sensor still needs to be improved. In the future work, the filter and amplifier can be used to improve the SNR, so as to further improve the performance of the sensor. The sensor has the advantages of simple structure and low cost, which can be applied to the oil condition monitoring of equipment.

## Figures and Tables

**Table 1:** Detection results of the sensor without silicon steel strips.

Particle Type	Connection Mode	Inductance Pulse Amplitude/H	Inductance Signal Noise/H	Inductance SNR	Resistance Pulse Amplitude/Ω	Resistance Signal Noise/Ω	Resistance SNR
93 μm iron particle	Series connection	4.28 × 10^−9^	5.0 × 10^−10^	8.56		5.0 × 10^−3^	
	Parallel connection	9.4 × 10^−11^	3.0 × 10^−11^	3.13		3.0 × 10^−4^	
186 μm copper particle	Series connection	1.88 × 10^−9^	5.0 × 10^−10^	3.76	3.73 × 10^−2^	5.0 × 10^−3^	7.46
	Parallel connection	5.0 × 10^−11^	3.0 × 10^−11^	1.67	7.4 × 10^−4^	3.0 × 10^−4^	2.47

**Table 2:** Detection results of the sensor with silicon steel strips.

Particle Type	Connection Mode	Inductance Pulse Amplitude/H	Inductance Signal Noise/H	Inductance SNR	Resistance Pulse Amplitude/Ω	Resistance Signal Noise/Ω	Resistance SNR
93 μm iron particle	Series connection	7.64 × 10^−9^	5.0 × 10^−10^	15.28	1.65 × 10^−2^	5.0 × 10^−3^	3.30
	Parallel connection	1.72 × 10^−10^	3.0 × 10^−11^	5.73	4.7 × 10^−4^	3.0 × 10^−4^	1.57
186 μm copper particle	Series connection	5.63 × 10^−9^	5.0 × 10^−10^	11.26	8.03 × 10^−2^	5.0 × 10^−3^	16.06
	Parallel connection	1.05 × 10^−10^	3.0 × 10^−11^	3.50	1.69 × 10^−3^	3.0 × 10^−4^	5.63

## Data Availability

Data is contained within the article.
